# Toxicokinetics of β-Amanitin in Mice and In Vitro Drug–Drug Interaction Potential

**DOI:** 10.3390/pharmaceutics14040774

**Published:** 2022-04-01

**Authors:** Young Yoon Bang, Im-Sook Song, Min Seo Lee, Chang Ho Lim, Yong-Yeon Cho, Joo Young Lee, Han Chang Kang, Hye Suk Lee

**Affiliations:** 1 College of Pharmacy and BK21 Four-Sponsored Advanced Program for SmartPharma Leaders, The Catholic University of Korea, Bucheon 14662, Korea; js7123258@catholic.ac.kr (Y.Y.B.); minseo.lee@catholic.ac.kr (M.S.L.); maxlim8580@catholic.ac.kr (C.H.L.); yongyeon@catholic.ac.kr (Y.-Y.C.); joolee@catholic.ac.kr (J.Y.L.); hckang@catholic.ac.kr (H.C.K.); 2 BK21 FOUR Community-Based Intelligent Novel Drug Discovery Education Unit, Vessel-Organ Interaction Research Center (VOICE), College of Pharmacy and Research Institute of Pharmaceutical Sciences, Kyungpook National University, Daegu 41566, Korea; isssong@knu.ac.kr

**Keywords:** β-amanitin, mouse, toxicokinetics, tissue distribution, drug interaction, drug-metabolizing enzymes, drug transporters

## Abstract

The toxicokinetics of β-amanitin, a toxic bicyclic octapeptide present abundantly in Amanitaceae mushrooms, was evaluated in mice after intravenous (iv) and oral administration. The area under plasma concentration curves (AUC) following iv injection increased in proportion to doses of 0.2, 0.4, and 0.8 mg/kg. β-amanitin disappeared rapidly from plasma with a half-life of 18.3–33.6 min, and 52.3% of the iv dose was recovered as a parent form. After oral administration, the AUC again increased in proportion with doses of 2, 5, and 10 mg/kg. Absolute bioavailability was 7.3–9.4%, which resulted in 72.4% of fecal recovery from orally administered β-amanitin. Tissue-to-plasma AUC ratios of orally administered β-amanitin were the highest in the intestine and stomach. It also readily distributed to kidney > spleen > lung > liver ≈ heart. Distribution to intestines, kidneys, and the liver is in agreement with previously reported target organs after acute amatoxin poisoning. In addition, β-amanitin weakly or negligibly inhibited major cytochrome P450 and 5′-diphospho-glucuronosyltransferase activities in human liver microsomes and suppressed drug transport functions in mammalian cells that overexpress transporters, suggesting the remote drug interaction potentials caused by β-amanitin exposure.

## 1. Introduction

Amatoxins are highly toxic bicyclic octapeptides found in mushrooms, such as *Amanita phalloides*. These peptides include α-, β-, γ-, and ε-amanitin, amanullin, amanullinic acid, amaninamide, amanin, phallaloidin, and phallacidin [1,2,3,4]. The toxic effects of phallatoxins are known to be limited, but α- and β-amanitin are the major amatoxins in poisonous mushrooms [5]. These substances cause poisoning by inhibiting RNA polymerase II, DNA transcription, and protein synthesis in eukaryotic cells [6,7,8,9,10,11]. Amatoxins are stable during cooking, drying, and other processing steps [12]. These chemicals are heat-stable, water-soluble, and resistant to enzyme and acid degradation [13,14]. In addition, amatoxins decompose slowly when stored in an aqueous solution or when exposed to ultraviolet (UV) light for long periods [1,2,15,16], which could potentiate the toxicity of amatoxins upon exposure in vivo.

*Amanita phalloides* poisoning can cause acute hepatitis, leading to the rapid development of liver insufficiency and ultimately coma and death [2,13]. Nephrotoxicity is reported less frequently [17]. Yilmaz et al. [7] reported that an oral intake of approximately 50 g of fresh *Amanita phalloides*, representing a dose of 0.32 mg/kg of amatoxins, can be lethal. A few reports are available that describe the toxicokinetics of α- and β-amanitin after the oral administration of toadstool extract to beagle dogs and rats [9,18,19]. In addition, the toxicokinetics of α-amanitin were investigated after intravenous (iv), intraperitoneal, and oral administration of amatoxin in rats and mice [14,18,20,21]. Low absolute bioavailability (3.5–4.8%) and substantial distribution to the intestines, kidneys, and liver were observed after the oral administration of α-amanitin at doses of 2, 5, or 10 mg/kg to mice [14]. Bang et al. [22] developed a sensitive analytical method for the simultaneous determination of α- and β-amanitin in mouse plasma using high-resolution mass spectrometry (LC-HRMS) and measured plasma concentrations of α- and β-amanitin after the oral administration of α- and β-amanitin at 1.5 mg/kg dose in male ICR mice. α- and β-amanitin showed similar toxicokinetic profiles at low dose exposure [22].

In humans, the plasma and urinary concentrations of α- and β-amanitin were detected in cases of suspected amatoxin poisoning [23,24,25]. Among 43 amatoxin-intoxicated patients, amanitin was detected in 11 patients who were exposed to amatoxin within 36 h. The plasma concentrations were 8–190 ng/mL for α-amanitin and 15.9–162 ng/mL for β-amanitin [26]. The total amount of α- and β-amanitin excreted in urine were measured in 24 patients out of 35 cases, and a highly variable amount of α- and β-amanitin was recovered from the urine: 0.03–3.29 mg for α-amanitin and 0.05–5.21 mg for β-amanitin [26]. Among 43 amatoxin-intoxicated patients, 4 cases had a result of α- and β-amanitin concentration in the liver and kidney: 10–19 ng/g liver and 122–1719 ng/g kidney for α-amanitin vs. 170.8–3298 ng/g liver and 1017–1391 ng/g kidney for β-amanitin [26]. The results indicated the possibility of higher tissue concentrations of β-amanitin than α-amanitin despite the similar plasma concentrations of α- and β-amanitin. It raised the different toxicokinetic properties and tissue distribution profiles of α- and β-amanitin.

**Figure 1:**
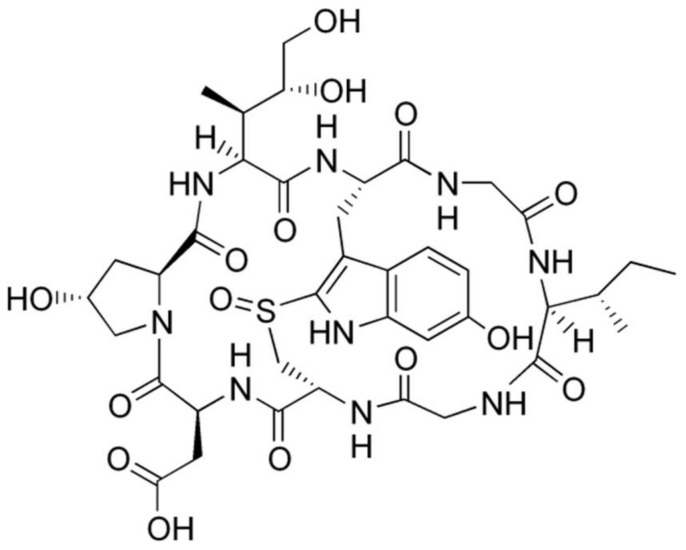
The chemical structure of β-amanitin.

In addition, bile drainage caused more than a 70% reduction in plasma concentration of α- and β-amanitin and reduced hepatic toxicity following the oral administration of dried powder of *Amanita exitialis* (60 mg/kg) [19]. The results suggested that biliary excretion of α- and β-amanitin plays a crucial role in the toxicokinetics of these toxins, and the contribution of biliary excretions of α- and β-amanitin to their absorption may be crucial to understanding their toxicokinetics as well as hepatic toxicity. However, the toxicokinetics and tissue distribution of β-amanitin (Figure 1) upon exposure to different doses and different routes of administration remain unclear. Thus, this study aimed to investigate the toxicokinetic profile of β-amanitin after its oral and intravenous administration with various doses. To investigate the toxicokinetically susceptible tissues, we aimed to investigate the tissue distribution of β-amanitin in mice. Moreover, the involvement of hepatic and renal transporters, which are critical for excretion and tissue distribution of drugs [27,28,29], for α- and β-amanitin need to be investigated. Organic anion transporting polypeptides (OATP) 1B1 and OATP1B3 were selected as hepatic transporters that are exclusively expressed in the liver and are critical for liver distribution and biliary excretion of substrates [29]. Organic cation transporter (OCT)2, multidrug and toxin extrusion protein (MATE)2-K, organic anion transporter (OAT)1 and OAT3 were selected as renal transporters that are exclusively expressed in the kidney and regulate kidney distribution and urinary excretion of substrates [29]. In addition, the interactions of β-amanitin with hepatic and renal transporters could increase susceptibility to β-amanitin and cause toxin–drug interactions, similar to the interaction between OATP1B3 and α-amanitin [30]. We also aimed to evaluate the in vitro inhibition of major cytochrome P450 (CYP) and 5′-diphospho-glucuronosyltransferase (UGT) enzymes by β-amanitin using human liver microsomes (HLMs) and on hepatic and renal transporters using a mammalian cell overexpression system [14,31,32].

## 2. Materials and Methods

### 2.1. Materials

β-amanitin (purity, 95.0%, Figure 1) was obtained from Cayman Chemical Co. (Ann Arbor, MI, USA). Acetaminophen, *N*-acetylserotonin, alamethicin, chenodeoxycholic acid, coumarin, dimethyl sulfoxide (DMSO), ketoconazole, meloxicam, mycophenolic acid, naloxone, naloxone 3-β-D-glucuronide, phenacetin, potassium phosphate dibasic trihydrate, potassium phosphate monobasic, SN-38, trifluoperazine, trifluoperazine *N*-β-D-glucuronide, Trizma Base, Trizma HCl, uridine 5′-diphosphoglucuronic acid (UDPGA), and reduced β-nicotinamide adenine dinucleotide phosphate tetrasodium salt (NADPH), cimetidine, probenecid, rifampin, Hank’s balanced salt solution (HBSS), sodium butyrate, and non-essential amino acids were purchased at Sigma-Aldrich Co. (St. Louis, MO, USA). *N*-acetylserotonin β-D-glucuronide, amodiaquine, bupropion, chenodeoxycholic acid 24-acyl-β-glucuronide, *N*-deethylamodiaquine, diclofenac, 4′-hydroxybupropion, 7-hydroxycoumarin, 4′-hydroxymephenytoin, mycophenolic acid β-D-glucuronide, propofol-β-D-glucuronide, SN-38 glucuronide, and [*S*]-mephenytoin were obtained from Toronto Research Chemicals Inc. (Toronto, ON, Canada). ^13^C_2_,^15^N-acetaminophen, bufuralol, 1′-hydroxybufuralol, 1′-hydroxymidazolam, 4′-hydroxydiclofenac, d_9_-1′-hydroxybufuralol, and ultrapooled HLMs (150 donors, mixed gender) were bought from Corning Life Sciences (Woburn, MA, USA). [^3^H]Para-aminohippuric acid (0.13 TBq/mmol), [^3^H]estrone-3-sulfate (2.12 TBq/mmol), [^3^H]estradiol-17β-D-glucuronide (2.22 TBq/mmol), [^3^H]taurocholate (0.57 TBq/mmol), and [^3^H]methyl-4-phenylpyridinium (2.9 TBq/mmol) were purchased from Perkin Elmer Inc. (Boston, MA, USA). [^14^C]Metformin (110 mCi/mmol) was purchased from Moravek (Brea, CA, USA). Formic acid was purchased from Honeywell (Charlotte, NC, USA). Acetonitrile, water, and methanol (LC-MS grade) were supplied from Fisher Scientific (Fair Lawn, NJ, USA). Other chemicals used the highest quality available. 

### 2.2. Toxicokinetic Study of β-Amanitin in Male ICR Mice

The Institutional Animal Care and Use Committee of the Catholic University of Korea (approval number, CUK-IACUC-2021-004; approval date, 21 March 2021) approved the study protocol. Male ICR mice (8 weeks of age, 27.4 ± 2.3 g) were purchased from Orient Bio Inc. (Seongnam, Korea). Mice were kept in plastic cages with unlimited access to a standard mouse diet (Orient Bio) and water before the experiments. For oral administration, mice were fasted for at least 12 h but allowed free access to water.

#### 2.2.1. Toxicokinetics Study

β-amanitin was dissolved in water and administered by iv bolus injection into the tail vein of mice at doses of 0.2 (*n* = 8), 0.4 (*n* = 6), and 0.8 (*n* = 7) mg/kg. Blood samples (approximately 15 μL) were collected from the retro-orbital plexus under light anesthesia with isoflurane at 0.033, 0.083, 0.25, 0.5, 0.75, 1, 1.5, 2, 3, 4, 6, and 8 h after drug administration.

β-amanitin was dissolved in water and administered to mice by gavage at doses of 2 (*n* = 7), 5 (*n* = 9), and 10 (*n* = 7) mg/kg. Blood samples (approximately 15 μL) were collected from the retro-orbital plexus under light anesthesia with isoflurane at 0.033, 0.083, 0.166, 0.25, 0.5, 0.75, 1, 1.5, 2, 4, 6, and 8 h after drug administration. Plasma samples (5 μL each) were harvested by centrifugation at 13,000× *g* for 3 min at 4 °C and stored at −80 °C until analysis.

#### 2.2.2. Tissue Distribution Experiments

Male mice fasted for 12 h with free access to water. Blood, brain, liver, kidney, heart, lung, stomach, intestine, and spleen were collected at 0.5, 1, 2, and 4 h after iv (0.8 mg/kg) injection or at 0.5, 1, 3, and 6 h after oral (10 mg/kg) administration of β-amanitin (*n* = 4 at each time point). Plasma samples (5 μL each) were harvested by centrifugation at 13,000× *g* for 3 min at 4 °C. Tissue samples were washed with cold normal saline, weighed, and stored at −80 °C until analysis.

#### 2.2.3. Excretion Experiments

β-amanitin in water was administered by iv bolus injection into the tail vein at 0.8 mg/kg dose (*n* = 4) and by oral gavage at 10 mg/kg dose (*n* = 3) to male mice. Mice were returned to metabolic cages, and urine and feces samples were collected individually at 0–6, 6–12, 12–24, 24–36, and 36–48 h. Urine and feces samples were stored at −80 °C until the analysis.

#### 2.2.4. Liquid Chromatography-High Resolution Mass Spectrometry (LC-HRMS) of β-Amanitin in Mouse Plasma, Tissue, Urine, and Feces Samples

Concentrations of β-amanitin in biological samples were evaluated following our previous methodology [22]. The standard calibration curves for β-amanitin in mouse plasma ranged from 0.5 to 500 ng/mL and were linear, with a correlation coefficient of 0.9952. The inter- and intra-day accuracy and precision fell within the acceptance criteria (3.1–14.6% of coefficients of variation (CVs) and 92.5–115.0% of accuracy). The extraction recovery and matrix effect of this analytical method were 82.8–88.9% and 93.0–98.6%. Three freeze–thaw cycles (88.7–97.2% accuracy with CVs of 3.6–14.3%), short-term storage for 2 h on ice (94.7–97.9% accuracy with CVs of 3.6–7.4%), long-term storage for 2 weeks at −80 °C (90.4–105.0% accuracy with CVs of 3.7–9.1%), and post-preparation stability for 24 h in 4 °C autosampler (90.7–94.1% accuracy with CVs of 6.8–7.5%) showed negligible effect on the stability of β-amanitin [22]. The standard calibration curves for β-amanitin in various tissue homogenates showed fairly good linearity and the intra-day accuracy and precision results from the quality control samples prepared in various tissue homogenates were all in acceptance criteria (Table S1, Supplementary Materials). 

An aliquot (5 μL) of plasma sample was mixed with 15 μL of 4′-hydroxydiclofenac (internal standard (IS), 5 ng/mL) in methanol and vortexed for 2 min. The mixture was centrifuged at 13,000× *g* at 4 °C for 5 min, and the supernatant was then transferred to an autosampler vial. An aliquot (5 μL) was injected into the LC-HRMS system for analysis.

Each tissue and feces sample was homogenized in water (1:3, *w*/*v*). In total, 50 μL aliquots of urine, tissue homogenates, or feces homogenates were mixed with 150 μL of 4′-hydroxydiclofenac solution (IS, 5 ng/mL in methanol). The other steps were as described for plasma sample preparation.

#### 2.2.5. Toxicokinetic Parameters and Statistical Analysis

The toxicokinetic parameters of β-amanitin were analyzed by non-compartmental analyses (WinNonlin, Pharsight; Mountain View, CA, USA) (Table 1). Data were expressed as mean ± standard deviations (SD). Comparisons of data between groups were performed using one-way ANOVA or Student *t*-test. Values of *p* < 0.05 were considered statistically significant.

### 2.3. Transport Studies of α- and β-Amanitin

HEK293 cells overexpressing OCT2, MATE2-K, OAT1, OAT3, OATP1B1, and OATP1B3 transporters and HEK293-control cells were maintained in a humidified atmosphere of 5% CO_2_ at 37 °C in DMEM supplemented with 10% FBS and 5 mM non-essential amino acids. In the case of HEK293-OATP1B1, -OATP1B3, and MATE2-K cells, 2 mM sodium butyrate was added to the culture medium to enhance transport activity [28,33]. Cells were seeded at 2 × 10^5^ cells/well in poly-D-lysine-coated 24-well plates. After 24 h, the growth medium was discarded, and attached cells were washed with HBSS and preincubated for 20 min in HBSS at 37 °C. Stock solutions of α- and β-amanitin and representative inhibitors of transporters were diluted in HBSS to make the final concentration: cimetidine 1 mM (for OCT2), cimetidine 100 μM (for MATE2-K), probenecid 100 μM (for OAT1 and OAT3), and rifampin 100 μM (for OATP1B1 and OATP1B3). Uptake of 50 μM α- and β-amanitin was measured in the absence and presence of representative inhibitors for 5 min at 37 °C. Plates were placed immediately on ice, and cells were then washed twice with 1 mL of ice-cold HBSS. Residual HBSS was removed thoroughly from the plates. Subsequently, 150 μL of 80% methanol containing IS was added to each sample well, and the cell plates were shaken gently for 20 min at 4 °C. The remaining steps were the same as described for plasma sample preparation.

### 2.4. Inhibitory Effects of β-Amanitin on the Major Drug-Metabolizing Enzymes and Transporters

β-amanitin inhibition (IC_50_ values) of CYP1A2, CYP2A6, CYP2B6, CYP2C8, CYP2C9, CYP2C19, CYP2D6, and CYP3A4 enzyme activities in HLMs were evaluated using LC-MS/MS and CYP substrates as previously described [31]. Each incubation mixture was made up to a volume of 100 μL including HLMs (0.2 mg/mL), 50 mM potassium phosphate buffer (pH 7.4), 1.0 mM NADPH, 10 mM magnesium chloride, various concentrations of β-amanitin (0.1–100 μM), a cocktail of seven CYP probe substrates (50 μM phenacetin for CYP1A2, 2.5 μM coumarin for CYP2A6, 2 μM amodiaquine for CYP2C8, 10 μM diclofenac for CYP2C9, 100 μM [*S*]-mephenytoin for CYP2C19, 5 μM bufuralol for CYP2D6, and 2.5 μM midazolam for CYP3A4 in acetonitrile), or 50 μM bupropion for CYP2B6. The reactions were initiated with NADPH followed by incubation in a shaking water bath at 37 °C for 15 min with or without 30 min preincubation with NADPH. Reactions were stopped with 50 μL of ice-cold methanol containing ^13^C_2_, ^15^N-acetaminophen and d_9_-1′-hydroxybufuralol (ISs). Incubation mixtures were centrifuged at 13,000× *g* for 8 min at 4 °C. An aliquot (5 μL) of the supernatant from the reaction mixture was analyzed by the previous LC-MS/MS method [31].

The inhibitory effect (IC_50_ values) of β-amanitin on UGT1A1, UGT1A3, UGT1A4, UGT1A6, UGT1A9, and UGT2B7 enzyme activities in ultrapooled HLMs was evaluated using two cocktails of UGT substrates and LC-MS/MS, as previously reported [32]. Each mixture was prepared to a final volume of 100 μL of ultrapooled HLMs (0.2 mg/mL), alamethicin (25 μg/mL), 50 mM Tris buffer (pH 7.4), 5 mM UDPGA, 10 mM magnesium chloride, various concentrations of β-amanitin (0.1–100 μM), and two sets of UGT enzyme-specific substrates in 50% methanol. Set A contained 0.5 μM SN-38 for UGT1A1, 2 μM chenodeoxycholic acid for UGT1A3, and 0.5 μM, trifluoperazine for UGT1A4; set B contained 1 μM *N*-acetylserotonin for UGT1A6, 0.2 μM, mycophenolic acid for UGT1A9, and 1 μM naloxone for UGT2B7). Reactions were initiated with the addition of UDPGA, then incubated in a shaking water bath at 37 °C for 60 min. The reactions were stopped with 50 μL of ice-cold acetonitrile containing ISs (propofol β-D-glucuronide for chenodeoxycholic acid 24-acyl-β-glucuronide and mycophenolic acid β-D-glucuronide and meloxicam for SN-38 glucuronide, trifluoperazine *N*-β-D-glucuronide, *N*-acetylserotonin β-D-glucuronide, and naloxone 3-β-D-glucuronide). After incubation, mixtures were centrifuged at 13,000× *g* for 8 min at 4 °C, and 50 μL of supernatants of sets A and B were mixed. An aliquot (2 μL) was analyzed using LC-MS/MS as previously described [32].

β-amanitin inhibition (IC_50_ values) of OCT1, OCT2, MATE2-K, OAT1, OAT3, Na^+^/taurocholate co-transporting polypeptide (NTCP), OATP1B1, OATP1B3, P-glycoprotein (P-gp), and breast-cancer-resistant protein (BCRP) transport activities were assessed in HEK293 cells overexpressing individual transporters (TransportoCells™, Corning), LLC-PK1-P-gp (Corning), or LLC-PK1-BCRP (obtained from Dr. A.H. Schinkel, Netherlands Cancer Institute; Amsterdam, The Netherlands), as previously reported [28,33]. Each incubation mixture was prepared to a final volume of 100 μL HBSS (pH 7.4) containing 0.1 μM probe substrates ([^3^H]methyl-4-phenylpyridinium for OCT1 and OCT2, [^14^C]metformin for MATE2-K, [^3^H]p-aminohippuric acid for OAT1, [^3^H]estrone-3-sulfate for OAT3, OATP1B1 and BCRP, [^3^H]taurocholate for NTCP, [^3^H]17-β-D-estradiol-glucuronide for OATP1B3, [^3^H]digoxin for P-gp) and various concentrations of β-amanitin (0.01–250 μM), as previously described [28,31,32,33].

## 3. Results

### 3.1. Toxicokinetics of β-Amanitin in Mice

First of all, intravenous bolus administration of 0.2, 0.4, and 0.8 mg/kg via tail vein and oral administration of 2, 5, and 10 mg/kg via oral gavage did not cause death or serious toxicity in mice in this study. 

**Figure 2:**
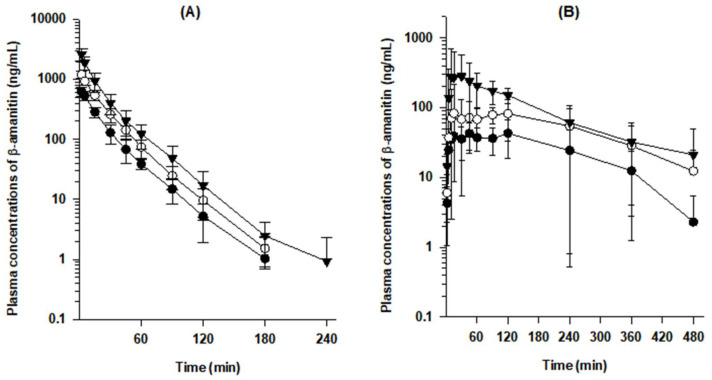
(**A**) Mean plasma concentration–time curves of β-amanitin in male ICR mice after an intravenous injection. ⬤: 0.2 mg/kg (*n* = 8); ◯: 0.4 mg/kg (*n* = 6); ▼: 0.8 mg/kg (*n* = 7). (**B**) Mean plasma concentration–time curves of β-amanitin in mice after oral administration. ⬤: 2 mg/kg (*n* = 7), ◯: 5 mg/kg (*n* = 9), and ▼: 10 mg/kg (*n* = 7). Each point represents mean ± SD.

**Figure 3:**
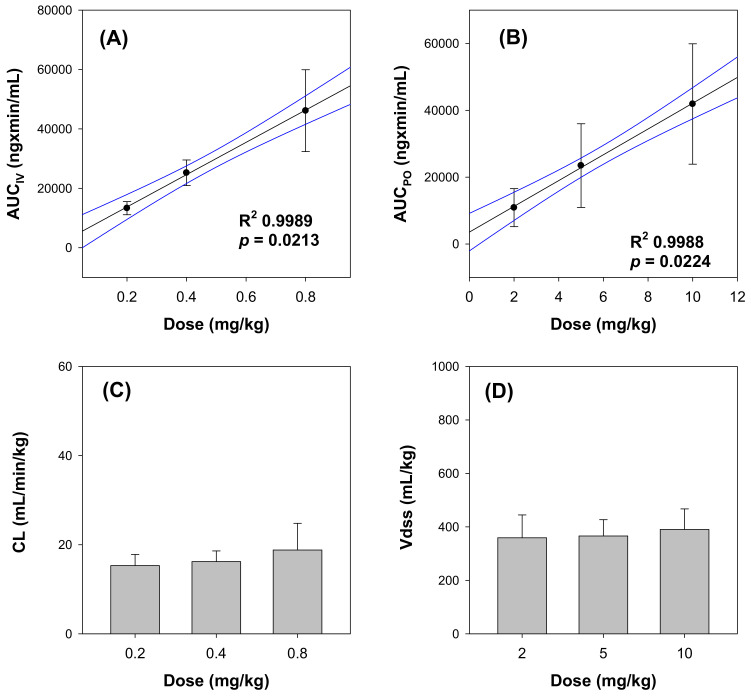
Correlations between AUC_IV_ (**A**) or AUC_PO_ (**B**) values for β-amanitin and intravenous or oral doses of β-amanitin. Lines were generated from linear regression analysis and 90% confidence intervals around the geometric mean value. R^2^ represents the correlation coefficient. (**C**) clearance (CL) and (**D**) volume of distribution (V_ss_) values of β-amanitin after iv bolus administration of β-amanitin were presented with β-amanitin doses. Each point represents mean ± SD.

Mean plasma concentration–time curves of β-amanitin after the iv administration of β-amanitin at doses of 0.2, 0.4, and 0.8 mg/kg demonstrated rapid elimination from plasma; t_1/2_ was in the range of 18.3–33.6 min (Figure 2A and Table 2). High systemic clearance (CL) after iv injection was observed for all three doses (Figure 2A and Table 2). Additionally, iv-injected β-amanitin showed linear kinetics in the dose range of 0.2–0.8 mg/kg, evidenced by a dose-proportional increase in AUC (Figure 3A) and dose-independent in CL and V_ss_ (Table 2 and Figure 3C,D). Consistent with the dose linearity of these parameters, the initial plasma concentration (C_0_) was also increased dose proportionally in the intravenous dose range of 0.2 ~–0.8 mg/kg. 

**Figure 4:**
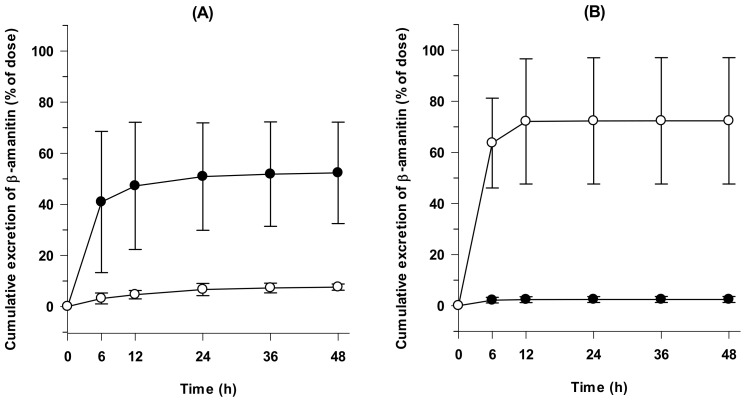
(**A**) Cumulative urinary and fecal excretion in mice after intravenous injection of β-amanitin (0.8 mg/kg) (*n* = 4). (**B**) Cumulative urinary and fecal excretion in mice after an oral administration of β-amanitin (10 mg/kg) (*n* = 3). ⬤: urine; ◯: feces. Each point represents mean ± SD.

The rapid elimination of β-amanitin from plasma was consistent with the prompt renal recovery of β-amanitin (Figure 4A). Total urinary recovery of β-amanitin after 48 h was 52.3 ± 19.8%, and 40.9% ± 27.6% was recovered within 6 h. Total fecal recovery of β-amanitin over 48 h was 7.6% ± 1.2%, much lower than urinary recovery (Figure 4A). More than 60% of iv-injected β-amanitin was recovered as a parent form, and it suggested the limited in vivo metabolism of β-amanitin.

Dose linearity was also observed after oral administration at doses of 2–10 mg/kg (Figure 2B and Figure 3B and Table 3). However, t_1/2_ values of orally administered β-amanitin (105.0~132.0 min) were much longer than iv-injected β-amanitin (18.3~33.6 min) (Table 2 and Table 3). Moreover, T_max_ showed a broad range from 10~120 min (Table 3). Thus, the absorption of β-amanitin likely occurred throughout the intestine, and delayed absorption may decrease the elimination rate of orally administered β-amanitin. Cumulative urinary and fecal excretion of β-amanitin for 48 h following oral administration was 2.4 ± 1.2% and 72.4 ± 24.7%, respectively (Figure 4B). These values also contrast the values measured after iv injection. Greater fecal recovery of β-amanitin could be attributed to unabsorbed compounds following oral administration, supporting the low estimate for oral bioavailability (i.e., 7.3–9.4%).

### 3.2. Tissue Distribution of β-Amanitin in Mice

**Figure 5:**
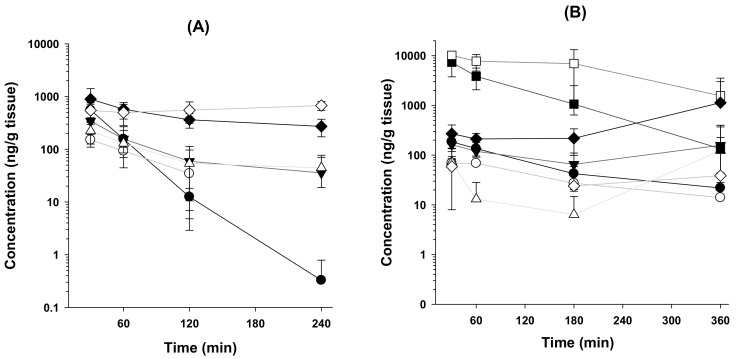
Mean concentration–time curves of β-amanitin in the plasma, heart, lung, liver, stomach, intestine, kidney, and spleen after (**A**) an intravenous injection (0.8 mg/kg) and (**B**) an oral administration (10 mg/kg). ⬤: plasma; ◯: heart; ▼: lung; △: liver; ◼: stomach; ☐: intestine; ◆: kidney; ◇: spleen. Each point represents mean ± SD (*n* = 4).

The tissue distribution of β-amanitin differs depending on the route of administration (Figure 5 and Table 4). Intravenously administered β-amanitin was distributed to the kidneys, spleen, heart, lungs, and liver and eliminated from these tissues at a slower rate compared to the elimination from plasma. Levels of β-amanitin in the brain, stomach, and intestines were below detection limits (Figure 5A), suggesting that the distribution of β-amanitin was restricted to some tissues. Tissue-to-plasma AUC ratios of β-amanitin in the heart, lungs, liver, spleen, and kidneys were 0.2, 0.7, 0.5, 2.6, and 2.5, respectively (Table 4). Conversely, tissue concentration of β-amanitin in the stomach, intestines, kidneys, lungs, and spleen was greater than that in plasma (Figure 5B), with high tissue-to-plasma AUC ratios of 28.8, 87.3, 6.7, 1.6, and 1.8, respectively (Table 4). These values in po administration were greater than those in iv injection. Tissue distribution to heart, lung, liver, and kidney was 0.5, 1.6, 0.5, and 1.8, respectively, which was similar to distribution after iv injection (Table 4). Thus, the stomach, intestine, and kidney may be target organs after oral β-amanitin poisoning. The heart, lung, liver, and spleen may also be target organs after β-amanitin exposure intravenously and orally in mice. In the case of the kidneys, liver, and spleen, tissue concentrations of orally administered β-amanitin tend to increase after 6 h, and it may cause delayed toxicity (Figure 5B).

### 3.3. Transport Substrate of α- and β-Amanitin for OATP1B1 and OATP1B3

**Figure 6:**
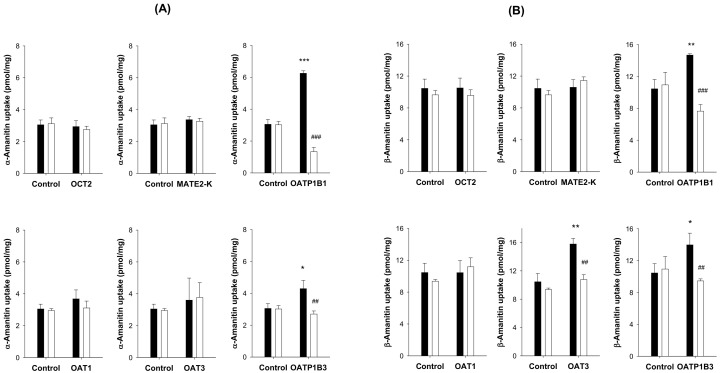
Uptake of (**A**) α-amanitin (50 μM) and (**B**) β-amanitin (50 μM) into HEK293 cells expressing renal transporters, such as OCT2, MATE2-K, OAT1, and OAT3, and hepatic transporters such as OATP1B1 and OATP1B3 transporters, respectively, was compared with uptake into HEK293 control cells. Uptake of α-amanitin or β-amanitin in the absence (◼) of representative inhibitors such as cimetidine 10 mM for OCT2, cimetidine 100 μM for MATE2-K, probenecid 200 μM for OAT1 and OAT3, and rifampin 100 μM for OATP1B1 and OATP1B3 was also compared with uptake in the presence of inhibitors (☐). Each data point represents the mean ± SD (*n* = 3). * *p* < 0.05; ** *p* < 0.01; *** *p* < 0.001 compared with control group. ## *p* < 0.01; ### *p* < 0.001 compared with inhibitor group.

Uptake of α- and β-amanitin into HEK293 cells expressing hepatic transporters, such as OATP1B1 and OATP1B3, was significantly increased compared to uptake into HEK293 control cells. Further, uptake was suppressed to control levels by the presence of rifampin, a representative inhibitor of OATP1B1 and OATP1B3 [34,35] (Figure 6). α- and β-amanitin are likely substrates for OATP1B1 and OATP1B3 and, therefore, are actively taken up into the liver. β-amanitin, but not α-amanitin, showed significantly greater uptake in HEK293-OAT3 compared with HEK293-control cells (Figure 6B). Uptake was suppressed to control levels by treatment of probenecid, a typical inhibitor of OAT3 [36]. Other renal transporters, such as OCT2, MATE2-K, and OAT1, were not involved in the active transport of α-amanitin or β-amanitin (Figure 6). Finally, uptake of β-amanitin into control cells was greater than α-amanitin. β-amanitin can more readily penetrate cell membranes, consistent with a previous report [37].

### 3.4. The Inhibitory Effects of β-Amanitin on the Major Drug-Metabolizing Enzymes and Transporters

**Figure 7:**
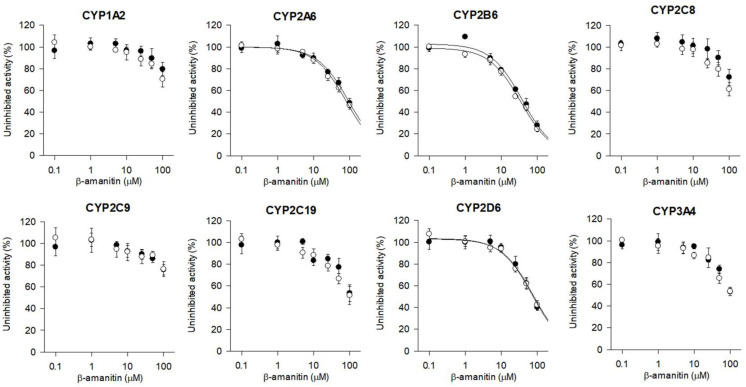
Inhibitory of eight major CYP enzyme activities by β-amanitin with (◯) and without (⬤) 30 min preincubation of NADPH in human liver microsomes. Phenacetin O-deethylation for CYP1A2, coumarin 7′-hydroxylation for CYP2A6, bupropion hydroxylation for CYP2B6, amodiaquine *N*-deethylation for CYP2C8, diclofenac 4′-hydroxylation for CYP2C9, [*S*]-mephenytoin 4′-hydroxylation for CYP2C19, bufuralol 1′-hydroxylation for CYP2D6, and midazolam 1′-hydroxylation for CYP3A4 were measured by LC-MS/MS. Each data point represents mean ± SD (*n* = 3).

The inhibition of eight major CYP and six major UGT enzyme activities by β-amanitin was evaluated in HLMs. β-amanitin weakly inhibited CYP2A6-catalyzed coumarin 7′-hydroxylation (IC_50_, 93.9 μM), CYP2B6-catalyzed bupropion hydroxylation (IC_50_, 38.0 μM), and CYP2D6-catalyzed bufuralol 1′-hydroxylation (IC_50_, 76.2 μM) in HLMs. β-amanitin at concentrations up to 100 μM showed negligible inhibition of CYP1A2-catalyzed phenacetin *O*-deethylation, CYP2C8-catalyzed amodiaquine *N*-deethylation, CYP2C9-catalyzed diclofenac 4′-hydroxylation, CYP2C19-mediated [*S*]-mephenytoin hydroxylation, and CYP3A4-mediated midazolam hydroxylation of β-amanitin in HLMs (Figure 7). No time-dependent inhibition of eight CYPs by β-amanitin after 30 min of preincubation was observed (Figure 7, Table 5).

**Figure 8:**
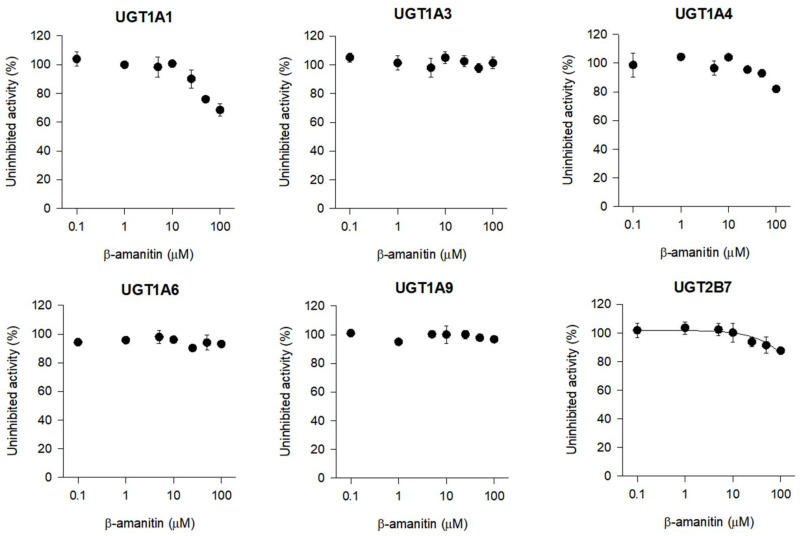
Inhibition of UGT1A1-catalyzed SN-38 glucuronidation, UGT1A3-catalyzed chenodeoxycholic acid 24-acyl-glucuronidation, UGT1A4-catalyzed trifluoperazine *N*-glucuronidation, UGT1A6-catalyzed *N*-acetylserotonin glucuronidation, UGT1A9-catalyzed mycophenolic acid glucuronidation, and UGT2B7-catalyzed naloxone 3-β-D-glucuronidation by β-amanitin in human liver microsomes using cocktail substrates and LC-MS/MS. Each data point represents mean ± SD (*n* = 3).

β-amanitin did not inhibit UGT1A1-catalyzed SN-38 glucuronidation, UGT1A3-catalyzed chenodeoxycholic acid 24-acyl-β-glucuronidation, UGT1A4-catalyzed trifluoperazine *N*-β-D-glucuronidation, UGT1A6-catalyzed *N*-acetylserotonin β-D-glucuronidation, UGT1A9-catalyzed mycophenolic acid β-D-glucuronidation, or UGT2B7-catalyzed naloxone 3-β-D-glucuronidation at 100 μM in HLMs (Figure 8).

**Figure 9:**
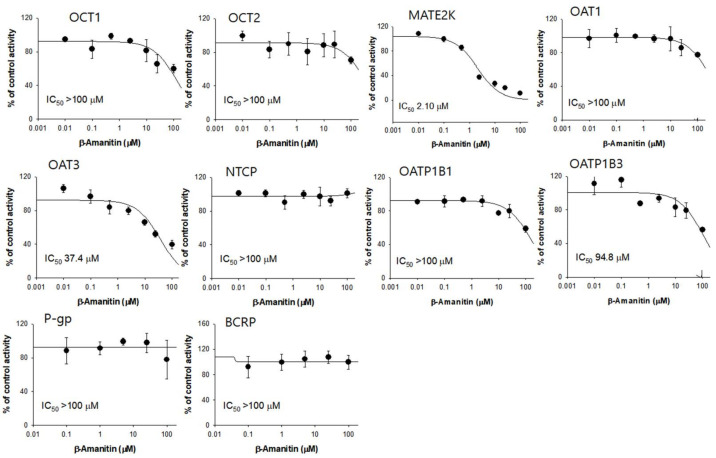
Inhibition of ten major transporters by β-amanitin in HEK293 or LLC-PK1 cells overexpressing respective transporters. OCT1- or OCT2-mediated [^3^H]methyl-4-phenylpyridinium uptake in HEK293-OCT1 or -OCT2 cells, MATE2-K-mediated [^14^C]metformin uptake in HEK293-MATE2-K cells, OAT1-mediated [^3^H]p-aminohippuric acid uptake in HEK-293-OAT1 cells, NTCP-mediated [^3^H]taurocholate uptake into HEK293-NTCP cells, OAT3- or OATP1B1-mediated [^3^H]estrone-3-sulfate uptake in HEK293-OAT3 or –OATP1B1 cells, OATP1B3-mediated [^3^H]17-β-D-estradiol-glucuronide in HEK293-OATP1B3 cells, P-gp-mediated [^3^H]digoxin transport in LLC-PK1-MDR1 cells, and BCRP-mediated [^3^H]estrone-3-sulfate transport in LLC-PK1-BCRP cells were measured. Each data point represents mean ± SD (*n* = 3).

We also evaluated the modulation of drug transporters by β-amanitin using an uptake and efflux transporter overexpression system. A weak or negligible inhibitory effect of β-amanitin on hepatic uptake transport activities of OCT1, NTCP, OATP1B1, and OATP1B3 was observed in the concentration range of 0.1–100 μM (Figure 9). In contrast, β-amanitin inhibited renal transport by MATE2-K and OAT3 in a concentration-dependent manner, yielding IC_50_ values of 2.1 μM and 37.4 μM, respectively. Other renal transporters OCT3 and OAT1 were not inhibited by the presence of β-amanitin in the concentration range of 0.1–100 μM (Figure 9). Efflux transporters, such as P-gp and BCRP, which often serve as absorption barriers to xenobiotics [33], were also not modulated by the presence of β-amanitin (Figure 9).

## 4. Discussion

The Amanitaceae family is responsible for nearly 95% of all fatal mushroom poisonings, and amatoxins are the primary threats to human life [7]. The content of amatoxins varies by Amanita species, but α- and β-amanitin are the most abundant substances. For example, an amatoxin content of 9.3 mg/g was observed in dried mushrooms and α- and β-amanitin accounted for 56% of toxins found in dried powder from *Amanita phalloides* [5]. α- and β-amanitin account for 82% of toxins (2.87 mg of amanitin/3.49 mg peptide toxins/g dried powder) in *Amanita exitialis* [9,19]. Among amatoxins, α-amanitin is of interest for toxicological investigation. The lethal dose of α-amanitin is reported to be 0.3–0.6 mg/kg in mice and 4.0 mg/kg in rats following intraperitoneal injection, and 0.1 mg/kg in humans after oral administration [2,20,38]. However, little information regarding toxicokinetics, tissue distribution, and toxic doses of β-amanitin is available in the literature. Bolus administration of 0.8 mg/kg via tail vein and 10 mg/kg via oral gavage did not cause toxicity in the present study.

β-amanitin showed a short t_1/2_ and high CL for all iv doses (Table 2). The rapid elimination of β-amanitin in the urine in parent form is likely the explanation (Figure 3B). Sun et al. reported that α-amanitin, excreted through glomerular filtration, was reabsorbed in renal tubules and caused kidney toxicity [20,39]. In addition, α-amanitin is actively taken up into the liver via hepatic transporters, OATP1B1 and OATP1B3 (Figure 6), which could lead to hepatotoxicity [10,26]. Similar to the case of α-amanitin, accumulation of β-amanitin in the liver and kidneys might also correlate with liver and kidney toxicity. OATP1B1- and OATP1B3-mediated hepatic uptake and OAT3-mediated kidney uptake of β-amanitin (Figure 6), along with reabsorption into renal tubules, may be involved. The potency of α-amanitin was 10-fold greater than β-amanitin in MCF-7 cells [37]. Thus, in spite of approximately two-fold higher cellular uptake mediated by hepatic or renal transporters and higher liver or kidney accumulation of β-amanitin, the contribution of β-amanitin to in vivo amatoxin toxicity may be lower than α-amanitin. α- and β-amanitin share common features in the elimination process, including major elimination in the urine without significant metabolism [11,40]. This similarity likely reflects their structural similarity. The only difference in structure is a -NH_2_ group in α-amanitin vs. an -OH group in β-amanitin. The hydroxyl group confers greater affinity for OAT3, higher cellular penetration, and higher accumulation in the heart, kidney, and spleen.

Interestingly, orally administered α- and β-amanitin showed distinctive accumulation in the stomach and intestine ([14] and Table 4 in this study), leading to prolonged absorption of toxicity and, consequently, a delay in elimination and potentiation of toxicity. Sun et al. [19] showed that the interruption of enterohepatic cycling of amatoxins by biliary drainage in dogs induced a 70% reduction in intestinal amatoxin absorption and lessened signs of severe toxicity. Taken together, intestinal absorption of and exposure to amatoxin may correlate with toxicity and could underlie different amatoxin toxicities depending on the route of poisoning. In this study, concentrations of orally administered β-amanitin in the kidney, liver, and spleen tend to increase after 6 h. This could lead to delayed toxicity in these tissues, which is consistent with the latent period of hepatic and kidney toxicity [41].

The intestinal tract, liver, and kidney are vulnerable to amanitin toxicity [2,13] and also express drug-metabolizing enzymes and transporters that regulate the absorption, distribution, metabolism, and excretion of endogenous substrates and xenobiotics [30,42]. In vitro inhibitory effects of β-amanitin on major human drug-metabolizing enzymes and transporters were thus evaluated. β-amanitin showed weak inhibition of CYP2A6, CYP2B6, and CYP2D6 with IC_50_ values of 93.9, 38.0, and 76.2 μM, respectively. Inhibition of CYP1A2, CYP2C8, CYP2C9, CYP2C19, and CYP3A4 at 100 μM in HLMs was almost negligible (Figure 7). IC_50_ values of β-amanitin toward CYP2A6, CYP2B6, and CYP2D6 were much higher than concentrations observed (15.9 to 162 ng/mL) in a person suffering from poisoning [26]. Thus, a β-amanitin-induced drug interaction is not likely to be caused by CYP inhibition. β-amanitin inhibited MATE2-K and OAT3 with IC_50_ values of 2.1 and 37.4 μM, respectively. Again, these concentrations are much higher than the plasma concentrations found in a case of poisoning [26]. β-amanitin did not significantly inhibit other transport activities (i.e., OCT1, OCT2, OAT1, OATP1B1, OATP1B3, NTCP, P-gp, or BCRP) (Figure 9) and UGT enzyme activities (UGT1A1, UGT1A3, UGT1A4, UGT1A6, UGT1A9, or UGT2B7) (Figure 8). Thus, the possibility of drug interaction caused by β-amanitin poisoning is remotely related to drug-metabolizing enzymes and transporters, and these results also seem to be reassuring that α- and β-amanitin weakly or negligibly interacts with CYPs and UGTs, and drug transporters. In this study, β-amanitin is a substrate for OAT3, OATP1B1, and OATP1B3, and it inhibits the transport activity of MATE2-K and OAT3. The interaction between these transporters and β-amanitin could be caused by the direct interaction between β-amanitin and substrate probe drugs such as metformin, estrone-3-sulfate, and estradiol-17-β-D-glucuronide. After the 1 h incubation of β-amanitin and substrate probe drugs, the mass signal of β-amanitin was not affected by the presence of substrate drugs and vice versa (Figure S1, Supplementary Materials). The absence of interference between β-amanitin and substrate drugs indicated no significant chemical interaction between them.

In addition, the involvement of MATE2-K, OAT3, OATP1B1, and OATP1B3 could be used as the detoxification mechanism of β-amanitin, as the clinical therapy in amatoxin poisoning implies. Single or combined treatment of benzylpenicillin, silibinin, and *N*-acetylcysteine has been used [2]. The mechanism of these treatments may be attributed to the hepatoprotective and anti-oxidative effect of silibinin and *N*-acetylcysteine and the reduced hepatic distribution of α-amanitin by inhibiting OATP1B3 using benzyl penicillin and silibinin [2,30,43,44]. In this case, the detoxification mechanism of α-amanitin poisoning can be explained by inhibiting the interaction between α-amanitin and OATP1B3 rather than the direct interaction between a-amanitin and the treated drugs, such as benzylpenicillin, silibinin, and *N*-acetylcysteine.

In addition to the interaction between β-amanitin and drug transporters, further toxicokinetic features of β-amanitin after *iv* and oral dose escalation, including tissue distribution and excretion characteristics, as well as in vitro assessment of drug interactions with drug-metabolizing enzymes and transporters, would provide additional useful information for a safety assessment using an in vivo prediction model.

## 5. Conclusions

Intravenous and oral administration of β-amanitin suggests low bioavailability, high CL, and low V_ss_: The absorption of β-amanitin is limited, and excretion is rapid, mainly via the renal route. Tissue distribution studies showed that β-amanitin was accumulated in gastrointestinal tissues based on stomach- and intestine-to-plasma ratios and unabsorbed β-amanitin that was recovered in feces after oral administration. β-amanitin showed weak or negligible inhibition of major human CYP and UGT enzymes and drug transporters, suggesting the likelihood that β-amanitin causes drug interactions via inhibition of CYP and UGT enzymes and drug transporters is remote.

## Figures and Tables

**Table 1:** Toxicokinetic parameters and calculation method.

Parameters	Meaning	Calculation Method
Co	Initial plasma concentration estimated at time zero following iv injection	Intercept of the plasma concentration–time curve
K	Elimination constant	Slope of the terminal phase of the plasma concentration–time curve
AUC	Area under the plasma concentration–time curve	Linear trapezoidal method $\sum_{n=0}^{t}\left(c_{n}\;+\;c_{n+1}\right)\left(t_{n+1}\;-\;t_{n}\right)/2\;+\;C_{t}/k$)
T1/2	Elimination half-life	0.693/K
MRT	Mean residence time	AUMC (Area under the moment curve)/AUC
CL	Clearance	Dose/AUC
Vss	Volume of distribution at steady-state	CLxMRT
Cmax	Maximum plasma concentration	Graphical observation of the highest plasma concentration
Tmax	Time to reach Cmax	Graphical observation of time that showing the highest plasma concentration
F	Oral bioavailability	(Dose normalized AUCPO/Dose normalized AUCIV) × 100

**Table 2:** Toxicokinetic parameters of β-amanitin in male ICR mice after intravenous injection of β-amanitin at three doses (mean ± SD).

Parameters	0.2 mg/kg (*n* = 8)	0.4 mg/kg (*n* = 6)	0.8 mg/kg (*n* = 7)
C_0_ (ng/mL)	711.9 ± 182.3	1435.7 ± 324.2	3249.6 ± 903.6
AUC_last_ (ng⋅min/mL)	13,304.2 ± 2216.8	25,229.5 ± 4298.3	46,135.8 ± 13,772.3
AUC_inf_ (ng⋅min/mL)	13,366.7 ± 2203.5	25,281.4 ± 4285.2	46,253.2 ± 13,839.7
CL (mL/min/kg)	15.3 ± 2.5	16.2 ± 2.4	18.8 ± 6.0
V_ss_ (mL/kg)	359.3 ± 85.5	365.9 ± 61.0	390.1 ±77.1
t_1/2_ (min)	21.8 ± 8.3	18.3 ± 3.1	33.6 ± 22.4
MRT (min)	22.8 ± 3.5	22.4 ± 2.7	20.7 ± 3.5

**Table 3:** Toxicokinetic parameters of β-amanitin in male ICR mice after oral administration of three doses (mean ± SD).

Parameters	2 mg/kg (*n* = 7)	5 mg/kg (*n* = 9)	10 mg/kg (*n* = 7)
C_max_ (ng/mL)	67.1 ± 18.6	140.1 ± 115.8	360.1 ± 385.0
T_max_^a^ (min)	60 (10–120)	90 (15–120)	60 (10–120)
AUC_last_ (ng⋅min/mL)	10,875 ± 5688	23,467 ± 12,517	41,896 ± 18,014
AUC_inf_ (ng⋅min/mL)	11,433 ± 5770	26,266 ± 14,447	48,620 ± 23,655
t_1/2_ (min)	105.0 ± 94.1	132.0 ± 50.3	130.2 ± 78.5
F (%)	9.4 ± 4.9	8.1 ± 4.3	7.3 ± 3.1

^a^ T_max_ presented median values with the range in parentheses.

**Table 4:** The area under the plasma and tissue concentration–time curves (AUC_last_) for β-amanitin in male ICR mice after intravenous (iv) and oral (po) administration of β-amanitin at doses of 0.8 or 10 mg/kg, respectively, (mean ± SD) (*n* = 4).

Tissue	AUC_last_ of β-Amanitin (ng⋅min/mL Plasma; ng⋅min/g Tissue)		Tissue to Plasma Ratio	
	iv (0.8 mg/kg)	po (10 mg/kg)	iv	po
Plasma	54,606 ± 3464	23,667 ± 2812	-	-
Brain	-	-	-	-
Heart	13,397 ± 908	12,563 ± 2696	0.2	0.5
Lung	35,970 ± 3070	37,831 ± 11,434	0.7	1.6
Liver	25,780 ± 2440	12,665 ± 11,731	0.5	0.5
Stomach	-	680,749 ± 137,691	-	28.8
Intestine	-	2,066,298 ± 492,689	-	87.3
Spleen	137,855 ± 11,949	42,409 ± 33,867	2.5	1.8
Kidney	142,023 ± 7510	158,677 ± 84,620	2.6	6.7

-: not detected.

**Table 5:** Inhibition of eight major CYP enzyme activities by β-amanitin with and without a 30 min preincubation in the presence of NADPH in ultrapooled human liver microsomes.

CYPs	Enzyme Activities	IC_50_ (μM)	
		without NADPH Preincubation	with NADPH Preincubation
1A2	Phenacetin *O*-deethylase	>100	>100
2A6	Coumarin 7-hydroxylase	93.9	80.1
2B6	Bupropion hydroxylase	38.0	34.8
2C8	Amodiaquine *N*-deethylase	>100	>100
2C9	Diclofenac 4′-hydroxylase	>100	>100
2C19	[*S*]-Mephenytoin 4′-hydroxylase	>100	>100
2D6	Bufuralol 1′-hydroxylase	76.2	71.6
3A4	Midazolam 1′-hydroxylase	>100	>100

## Data Availability

Not applicable.

## Supplementary Materials

The following supporting information can be downloaded at: https://www.mdpi.com/article/10.3390/pharmaceutics14040774/s1, Figure S1: (A) Concentration of β-amanitin after incubating in the absence or presence of metformin, estrone-3-sulfate, and 17-β-D-estradiol-glucuronide in HBSS (pH 7.4) for 1 h. (B) The concentration of metformin, estrone-3-sulfate, and 17-β-D-estradiol-glucuronide after incubating in the absence or presence of β-amanitin in HBSS (pH 7.4) for 1 h. Each point represents mean ± SD (*n* = 4).; Table S1: Concentration ranges, representative regression equation, and correlation coefficients of the calibration curves and precision (coefficient of variation, CV) and accuracy values for β-amanitin in tissue homogenates. References [22,45,46,47] are cited in the Supplementary Materials.
