# Supplementary material for: Toxicokinetics of β-Amanitin in Mice and In Vitro Drug–Drug Interaction Potential
Source: Pharmaceutics. 2022 Apr 1;14(4):774. doi: 10.3390/pharmaceutics14040774 (PMC9030691; doi:10.3390/pharmaceutics14040774)
Supplement: Supplementary file 1 [file pharmaceutics-14-00774-s001.zip › pharmaceutics-1626327-supplementary.pdf]

pharmaceutics

Supplementary Materials

Toxicokinetics of β-Amanitin in Mice and In Vitro Drug–Drug Interaction Potential

Young Yoon Bang, Im-Sook Song, Min Seo Lee, Chang Ho Lim, Yong-Yeon Cho, Joo Young Lee, Han Chang Kang and Hye Suk Lee

1. Liquid chromatography-high resolution mass spectrometry (LC-HRMS) of β-amanitin in various tissue samples

Blood, brain, liver, kidney, heart, lung, stomach, intestine, and spleen tissues were collected, washed with cold normal saline, and weighted. Tissue samples were homogenized in water (1:3, w/v) and 50 µL aliquots of tissue homogenates were mixed with 150 µL of 4′-hydroxydiclofenac solution (IS, 5 ng/mL in methanol) and vortexed for 2 min. The mixture was centrifuged at 13,000 rpm at 4 °C for 5 min, and the supernatant was then transferred to an autosampler vial. An aliquot (5 µL) was injected into the LC-HRMS system for analysis, according to the previous methods [22]. The standard calibration curves for β-amanitin in various tissue homogenates were prepared individually using the respective blank tissue homogenates. Each calibration standard curve was validated using the quality control (QC) samples.

As results, the standard calibration curves for β-amanitin in various tissue homogenates showed fairly good linearity and the intra-day accuracy and precision results from the quality control samples prepared in various tissue homogenates were all in acceptance criteria (Supplementary Table 1).

Table S1. Concentration ranges, representative regression equation, and correlation coefficients of the calibration curves and precision (coefficient of variation, CV) and accuracy values for β-amanitin in tissue homogenates.

Tissue	Concentration range (ng/g tissue)	Representative regression equation	Correlation coefficient	QC (ng/g tissue)	Intra-day (n = 5)	
					Accuracy (%)	CV (%)
Kidney	25-5000	Y = -0.0016901+0.00028424X	0.9967	75, 750, 3750	94.3-110.6	3.6-8.3
Spleen	100-50000	Y = -0.016516+0.00022888X	0.9971	300, 3000, 37500	91.0-110.7	3.4-7.8
Liver	50-50000	Y = 0.015264+0.00028372X	0.9923	150, 3000, 37500	86.3-104.7	2.8-8.5
Intestine	100-50000	Y = -0.023386+0.00033329X	0.997	300, 15000, 37500	85.2-99.4	5.4-9.3
Stomach	100-50000	Y = -0.0051471+0.0002316X	0.9959	300, 15000, 37500	87.7-108.7	4.5-10.3
Lung	50-50000	Y = -0.016133+0.00032201X	0.9981	150, 3000, 37500	96.7-108.7	5.6-9.3
Heart	50-50000	Y = -0.016494+0.00033891X	0.9946	150, 3000, 37500	85.4-97.0	3.4-8.9
Brain	100-50000	Y = -0.014433+0.00021117X	0.9992	300, 15000, 37500	91.4-105.3	4.1-11.9

2. Interaction between β-amanitin and substrate probe drugs for MATE2-K, OAT2, OATP1B1, and OATP1B3

Each incubation mixture was prepared to a final volume of 1 mL HBSS (pH 7.4) containing 1 µM probe substrates (metformin for MATE2-K, estrone-3-sulfate for OAT3 and OATP1B1, 17-β-D-estradiol-glucuronide for OATP1B3) and 1 µM of β-amanitin and incubated at 37 °C for 1 h. Fifty µL aliquots of incubation mixture were mixed with 450 µL of IS solution (4′-hydroxydiclofenac 5 ng/mL in methanol for β-amanitin and propranolol 0.5 ng/mL in methanol for metformin, estrone-3-sulfate, and 17-β-D-estradiol-glucuronide)

and vortexed for 2 min. The mixture was centrifuged at 16,000 rpm at 4 °C for 5 min, and the supernatant was then transferred to an autosampler vial. An aliquot (5 µL) was injected into the LC-MS/MS system for analysis, according to the previous methods [22, 45-47].

As results, After the 1 h incubation of β-amanitin and substrate drugs, the mass signal of β-amanitin was not affected by the presence of substrate drugs (Supplementary Figure 1A). Similarly, mass signal of metformin, estrone-3-sulfate, and 17-β-D-estradiol-glucuronide was not changed by the incubation with β-amanitin (Supplementary Figure 1B). The absence of interference between β-amanitin and substrate drugs indicated no significant chemical interaction between them.

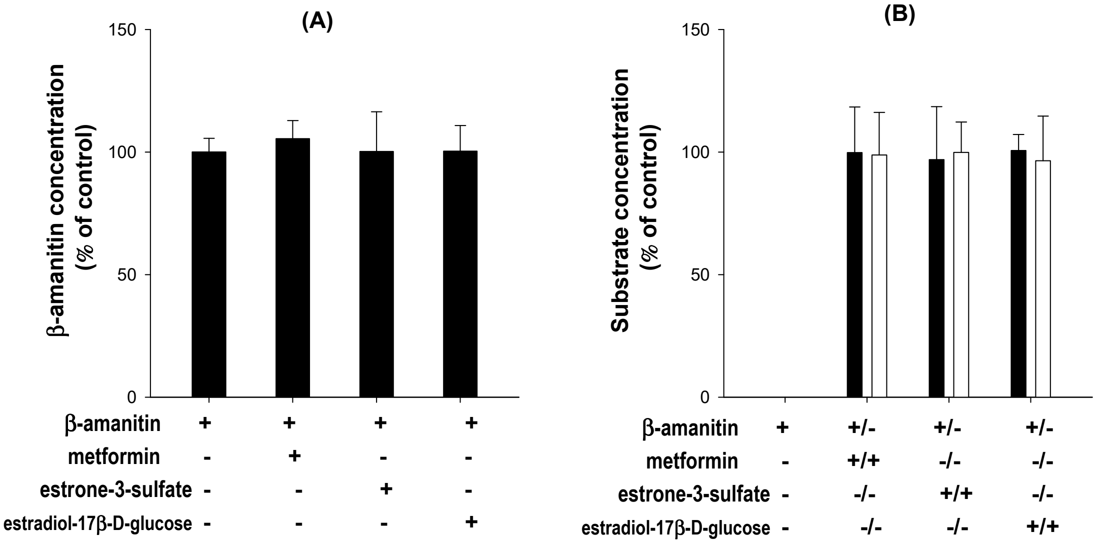

Figure S1. (**A**) Concentration of β-amanitin after incubating in the absence or presence of metformin, estrone-3-sulfate, and 17-β-D-estradiol-glucuronide in HBSS (pH 7.4) for 1 h. (**B**) Concentration of metformin, estrone-3-sulfate, and 17-β-D-estradiol-glucuronide after incubating in the absence or presence of β-amanitin in HBSS (pH 7.4) for 1 h. Each point represents mean ± SD (*n* = 4).
